# Supplementary material for: Extracellular thiamine concentration influences thermogenic competency of differentiating neck area-derived human adipocytes
Source: Front Nutr. 2023 Sep 13;10:1207394. doi: 10.3389/fnut.2023.1207394 (PMC10534038; doi:10.3389/fnut.2023.1207394)
Supplement: Supplementary file 1 [file Data_Sheet_1.pdf]

Supplementary Material

Extracellular thiamine concentration influences thermogenic competency of differentiating neck area-derived human adipocytes

Boglárka Ágnes Vinnai[1,2,†], Rini Arianti[1,3,†], Ferenc Győry[4], Zsolt Bacso[2,5,6], László Fésüs[1], Endre Kristóf[1,*]

*** Correspondence:** Endre Kristóf: kristof.endre@med.unideb.hu

1 Supplementary Figures and Tables

1.1 Supplementary Figures

a

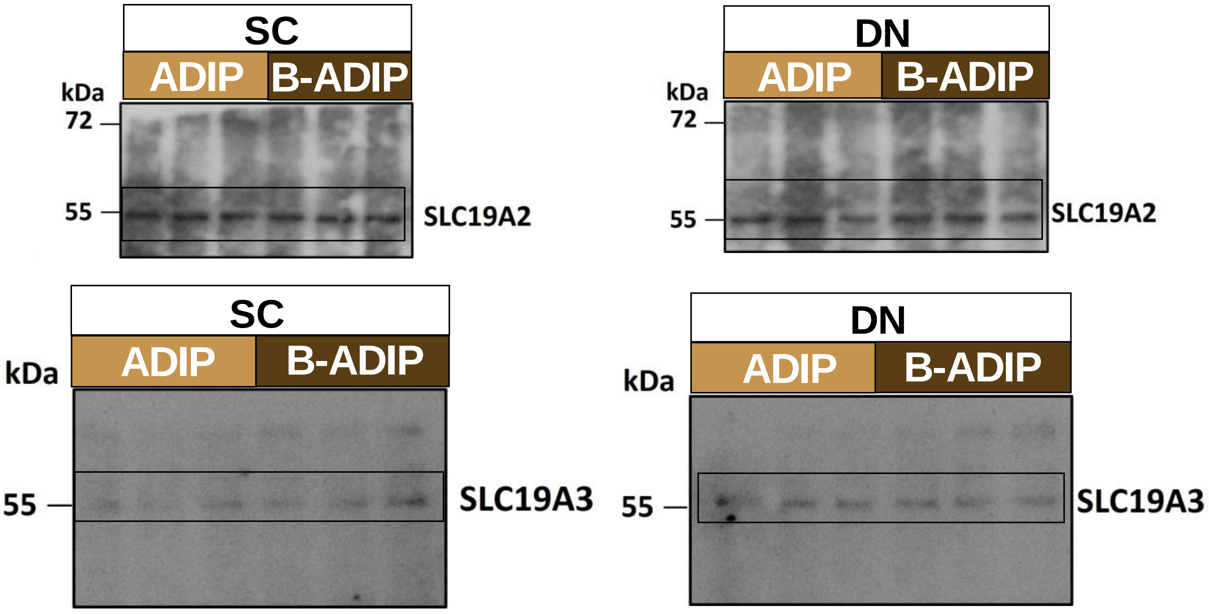
SC
ADIP
B-ADIP
kDa
72
55
SLC19A2
DN
ADIP
B-ADIP
kDa
72
55
SLC19A2
SC
ADIP
B-ADIP
kDa
55
SLC19A3
DN
ADIP
B-ADIP
kDa
55
SLC19A3

b

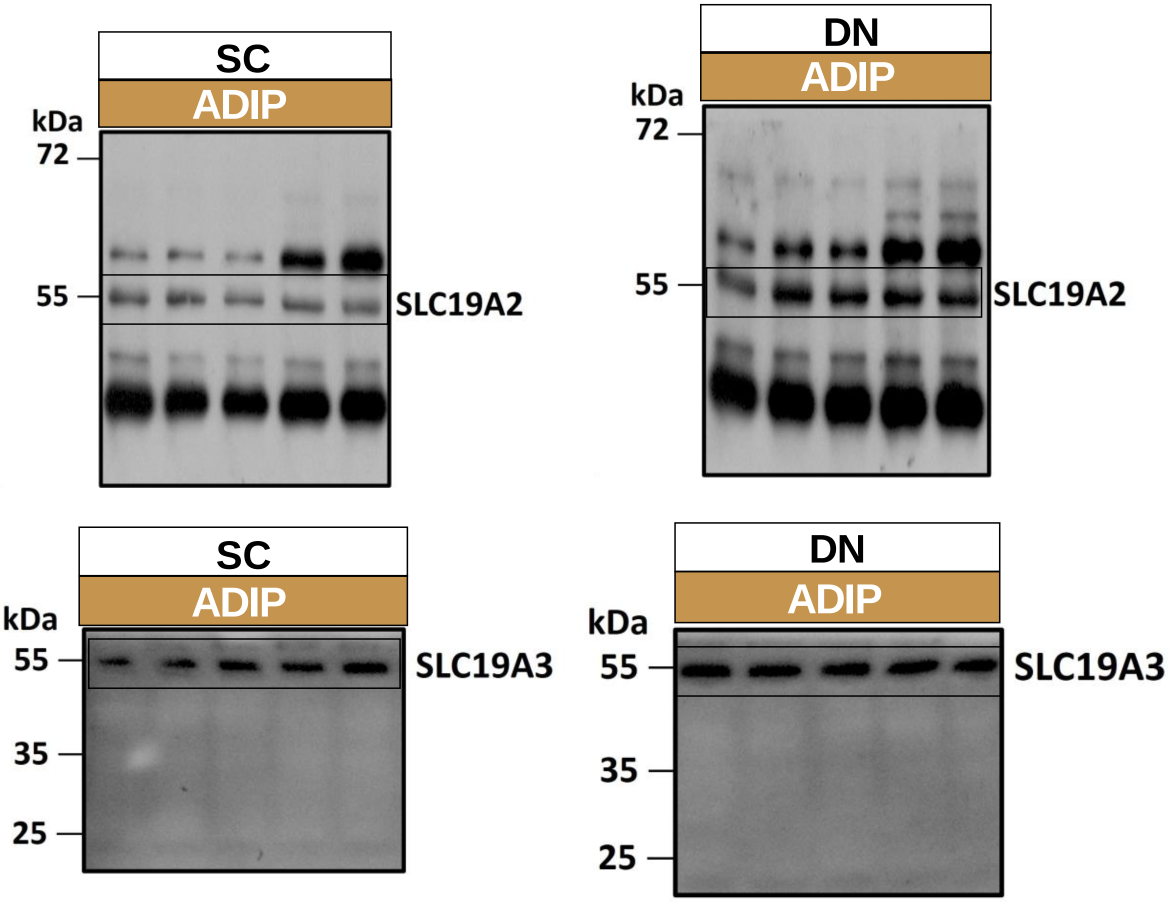
SC
ADIP
kDa
72
55
SLC19A2
DN
ADIP
kDa
72
55
SLC19A2
SC
ADIP
kDa
55
35
25
SLC19A3
DN
ADIP
kDa
55
35
25
SLC19A3

Supplementary Figure 1. Uncropped western blot images presented with molecular weight ladders, using polyclonal anti-SLC19A2 or anti-SLC19A3 antibodies as shown in Figure 1d (a) and Figure 2d (b). Tubulin was used as endogenous control. Cropped areas are shown in black box regions.

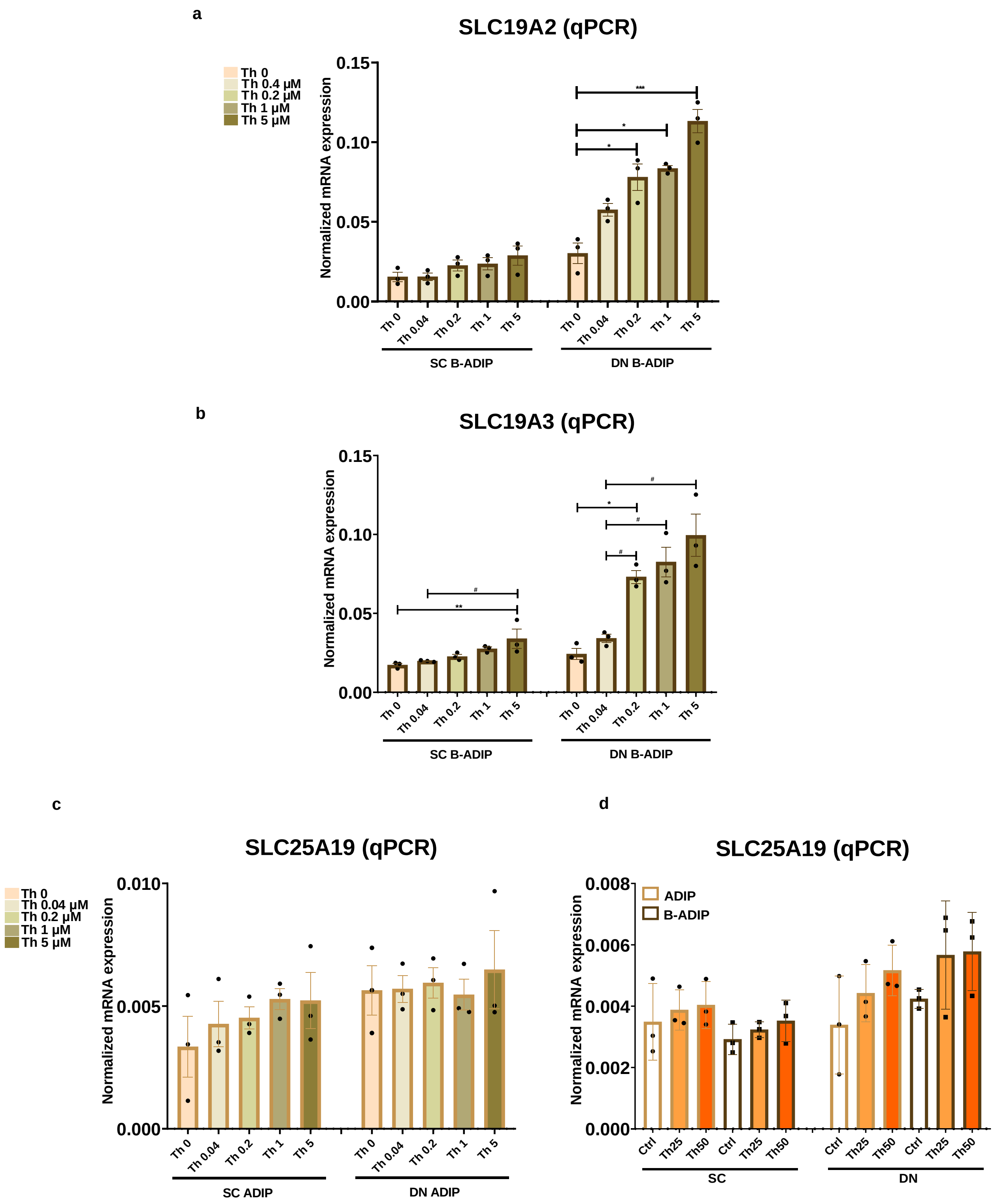

Supplementary Figure 2. Effect of gradually increasing concentrations (0.04 µM, 0.2 µM, 1 µM, 5 µM) of thiamine (Th) on the expression of Th transporters in human subcutaneous (SC) and deep neck (DN)-derived brown differentiated adipocytes (B-ADIPs). (a-b) mRNA expression of *SLC19A2* and *SLC19A3* assessed by RT-qPCR, n=3.

Effect of gradually increasing and excess (25 µM and 50 µM) concentrations of Th on the expression of mitochondrial Th pyrophosphate transporter (encoded by *SLC25A19*) in human SC and DN-derived adipocytes (ADIPs) and B-ADIPs. (c-d) mRNA expression of *SLC25A19* assessed by RT-qPCR, n=3. In case of the concentration-dependence experiments (a-c), statistical analysis was performed by one-way ANOVA. In case of experiments with excess thiamine (d), statistical analysis was performed by two-way ANOVA, *#p<0.05, **##p<0.01, ***###p<0.001, *comparing data at each concentration of Th to the lack of Th (Th 0) or # comparing the indicated groups.

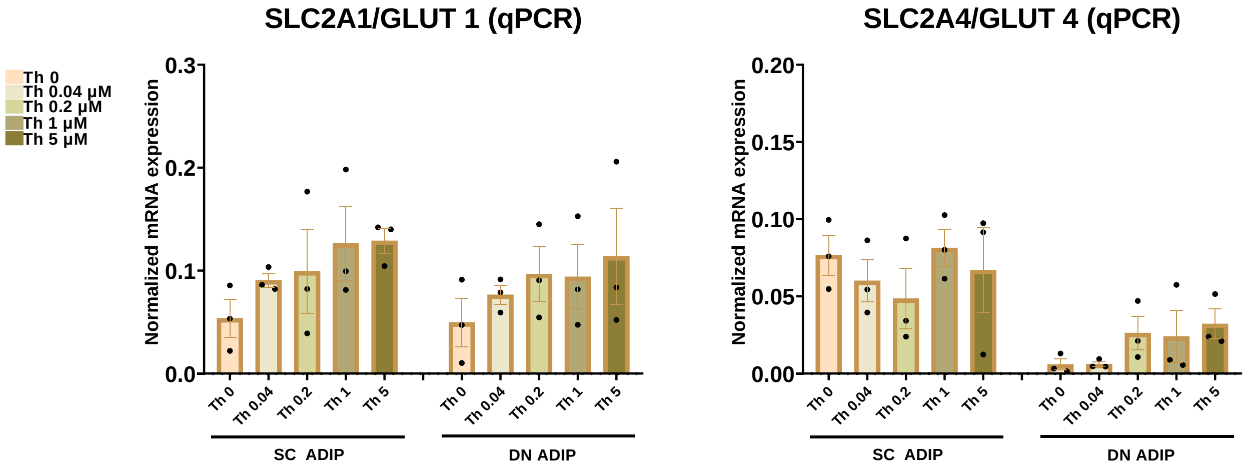

Supplementary Figure 3. Effect of gradually increasing concentrations of thiamine (Th) on the expression of GLUT transporters in human subcutaneous (SC) and deep neck (DN)-derived differentiated adipocytes (ADIPs). mRNA expression of *SLC2A1/GLUT1* and *SLC2A4/GLUT4* assessed by RT-qPCR, n=3. Statistical analysis was performed by one-way ANOVA.

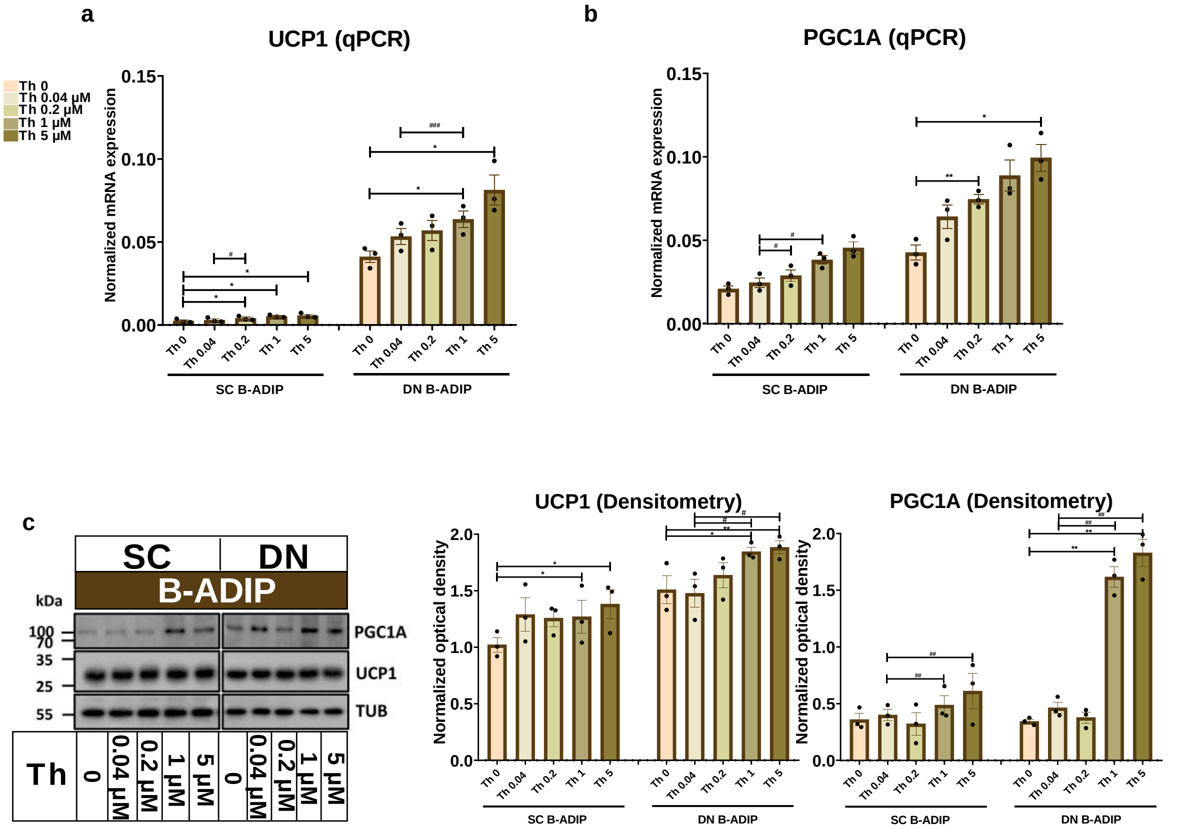

Supplementary Figure 4. Effect of gradually increasing concentrations of thiamine (Th) on thermogenic gene and protein expression in human subcutaneous (SC) and deep neck (DN)-derived brown differentiated adipocytes (B-ADIPs). (a-b) mRNA expression of *UCP1* and *PGC1a* assessed by RT-qPCR, n=3. (c) UCP1 and PGC1a protein expression detected by immunoblotting, n=3. Statistical analysis was performed by one-way ANOVA, *#p<0.05, **##p<0.01, ***###p<0.001, *comparing data at each concentration of Th to the lack of Th or # comparing the indicated groups.

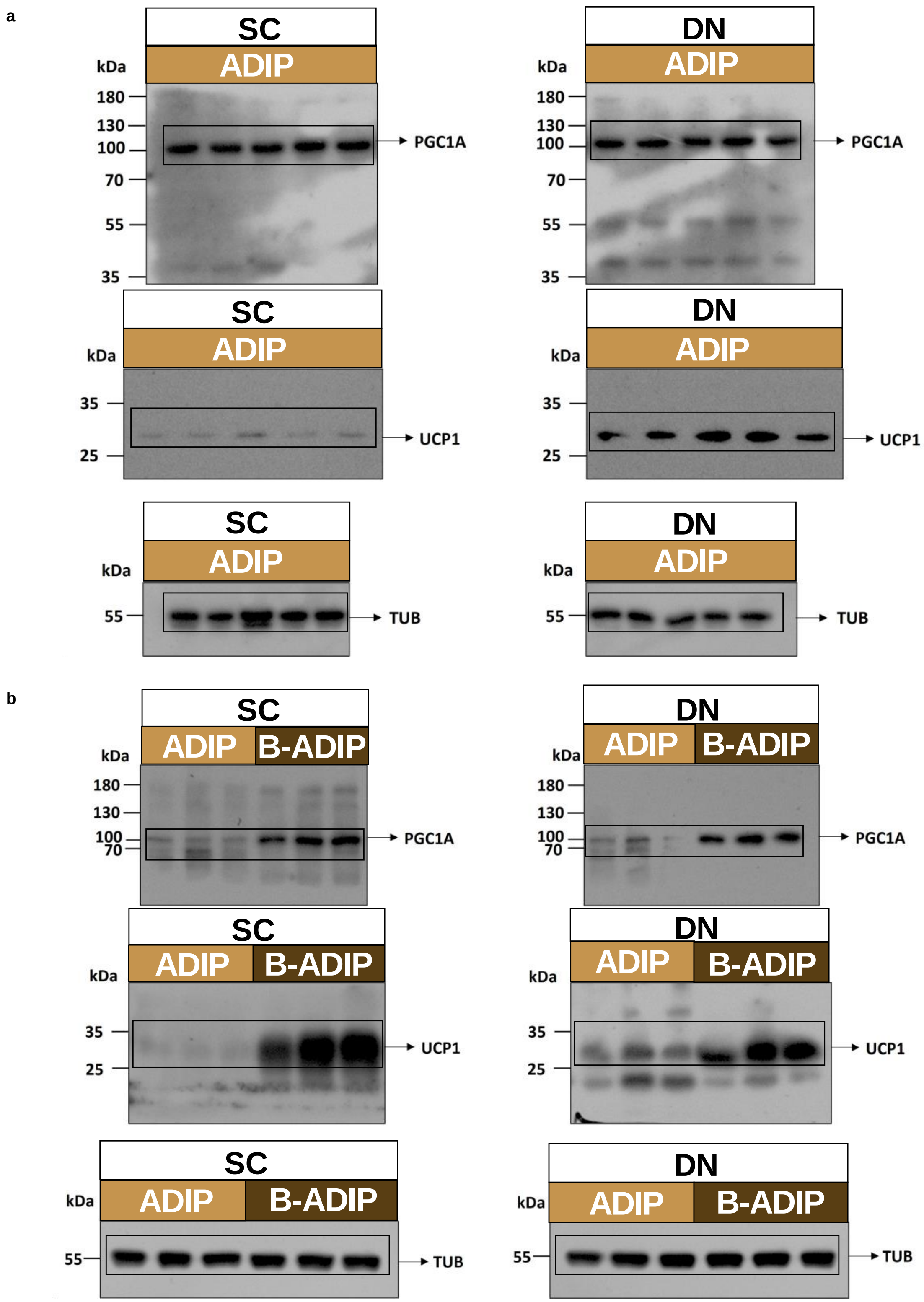

Supplementary Figure 5. Uncropped western blot images presented with molecular weight ladders, using MAB6158 monoclonal anti-UCP1 antibody or G0522 monoclonal anti-PGC1A antibody as shown in Figure 4c (a) and Figure 7c (b). Tubulin was used as endogenous control. Cropped areas are shown in black box regions.

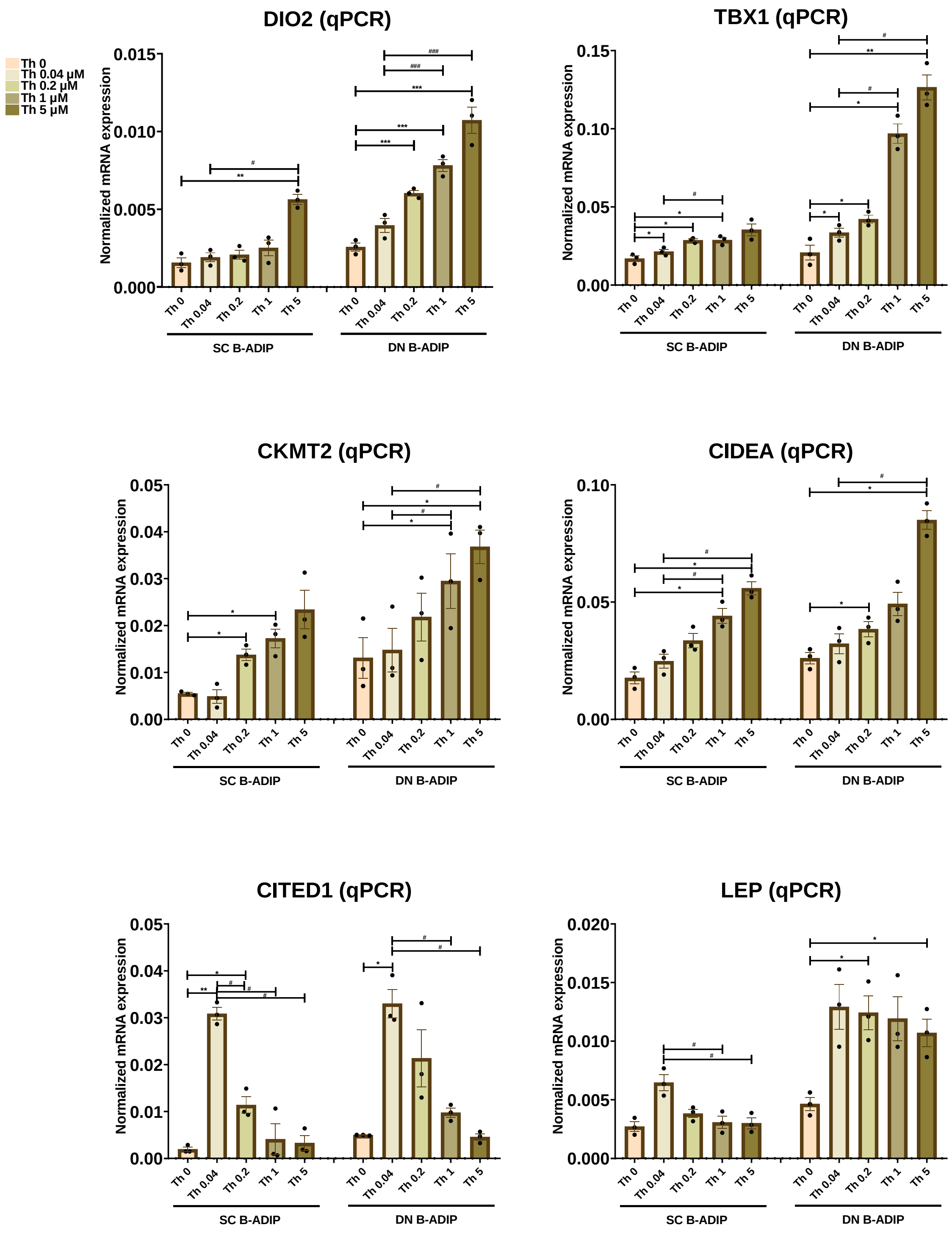

Supplementary Figure 6. Effect of gradually increasing concentrations of thiamine (Th) on thermogenic gene induction in human subcutaneous (SC) and deep neck (DN)-derived brown differentiated adipocytes (B-ADIPs). mRNA expression of *DIO2*, *TBX1*, *CKMT2*, *CIDEA*, *CITED1*, and *LEP* assessed by RT-qPCR, n=3. Statistical analysis was performed by one-way ANOVA, *#p<0.05, **##p<0.01, ***###p<0.001, *comparing data at each concentration of Th to the lack of Th (Th 0) or # comparing the indicated groups.

1.2 Supplementary Tables

Supplementary Table 1. Gene primers and probes

GENES	ASSAY ID
CIDEA	Hs00154455_m1
CITED1	Hs00918445_g1
CKMT2	Hs00176502_m1
DIO2	Hs00255341_m1
GAPDH	Hs99999905_m1
LEP	Hs00174877_m1
PPARGC1A	Hs01016719_m1
SLC19A2	Hs00949693_m1
SLC19A3	Hs00228858_m1
SLC2A1	Hs00892681_m1
SLC2A4	Hs00168966_m1
SLC25A19	Hs01001439_m1
TBX1	Hs00271949_m1
TMEM26	Hs00415619_m1
TNFRSF9	Hs00155512_m1
UCP1	Hs00222453_m1

Supplementary Table 2. Antibodies used in immunoblotting

ANTIBODY	COMPANY	CATALOG NUMBER	DILUTION
UCP1	R&D Systems, Minneapolis, MN, USA	MAB6158	1:750
SLC19A3	Novus Biologicals, Centennial, CO, USA	NBP1-69703	1:500
SLC19A2	Abcam, Cambridge, MA, USA	Ab229680	1:500
PGC1α	Novus Biologicals, Centennial, CO, USA	NBP1-04676	1:1000
Total OXPHOS	Abcam, Cambridge, MA, USA	ab110411	1:1000
TUBULIN	Santa Cruz, USA	sc-5274	1:10000
HRP-conjugated goat anti-rabbit IgG	Advansta, San Jose, CA, USA	R-05072-500	1:5000
HRP-conjugated goat anti-mouse IgG	Advansta, San Jose, CA, USA	R-05071-500	1:5000
